# Elucidation of Enzymatic Mechanism of Phenazine Biosynthetic Protein PhzF Using QM/MM and MD Simulations

**DOI:** 10.1371/journal.pone.0139081

**Published:** 2015-09-28

**Authors:** Fei Liu, Yi-Lei Zhao, Xiaolei Wang, Hongbo Hu, Huasong Peng, Wei Wang, Jing-Fang Wang, Xuehong Zhang

**Affiliations:** 1 State Key Laboratory of Microbial Metabolism, School of Life Sciences and Biotechnology, Shanghai Jiao Tong University, Shanghai, China; 2 Key Laboratory of Systems Biomedicine (Ministry of Education), Shanghai Center for Systems Biomedicine, Shanghai Jiao Tong University, Shanghai, China; Russian Academy of Sciences, Institute for Biological Instrumentation, RUSSIAN FEDERATION

## Abstract

The phenazine biosynthetic pathway is of considerable importance for the pharmaceutical industry. The pathway produces two products: phenazine-1,6-dicarboxylic acid and phenazine-1-carboxylic acid. PhzF is an isomerase that catalyzes *trans*-2,3-dihydro-3-hydroxyanthranilic acid isomerization and plays an essential role in the phenazine biosynthetic pathway. Although the PhzF crystal structure has been determined recently, an understanding of the detailed catalytic mechanism and the roles of key catalytic residues are still lacking. In this study, a computational strategy using a combination of molecular modeling, molecular dynamics simulations, and quantum mechanics/molecular mechanics simulations was used to elucidate these important issues. The Apo enzyme, enzyme–substrate complexes with negatively charged Glu45, enzyme–transition state analog inhibitor complexes with neutral Glu45, and enzyme–product complexes with negatively charged Glu45 structures were optimized and modeled using a 200 ns molecular dynamics simulation. Residues such as Gly73, His74, Asp208, Gly212, Ser213, and water, which play important roles in ligand binding and the isomerization reaction, were comprehensively investigated. Our results suggest that the Glu45 residue at the active site of PhzF acts as a general base/acid catalyst during proton transfer. This study provides new insights into the detailed catalytic mechanism of PhzF and the results have important implications for PhzF modification.

## Introduction

Phenazines are a large class of well-known natural and synthetic nitrogen-containing heterocyclic compounds. In the past century, more than 6000 compounds that contain the phenazine ring system have been identified, of which over 100 are natural derivatives isolated as secondary metabolites, mainly from microorganisms such as *Pseudomonas* spp. and *Streptomyces* spp. [1–8]. Most phenazines show broad-spectrum bioactivities such as antibiotic activities against bacteria, fungi, yeast, and parasites, and antitumor activities [1, 9–12]. Studies of the effects of phenazines have shown that these compounds have multiple functions, and not merely bioactivities. The results of a large number of studies of phenazines have shown that they play diverse roles in biofilm formation and architecture [13, 14], electron shuttles for modifying cellular redox states [15–17], cell signaling for regulating patterns of gene expression [18], and biotechnological applications such as pH indicators, biosensors, and fuel-cell components [19–21]. The importance of phenazines has attracted researchers in many disciplines such as biology, physiology, ecology, chemistry, pharmacy, and agriculture in recent years, and will continue to do so in the future.

**Fig 1:**
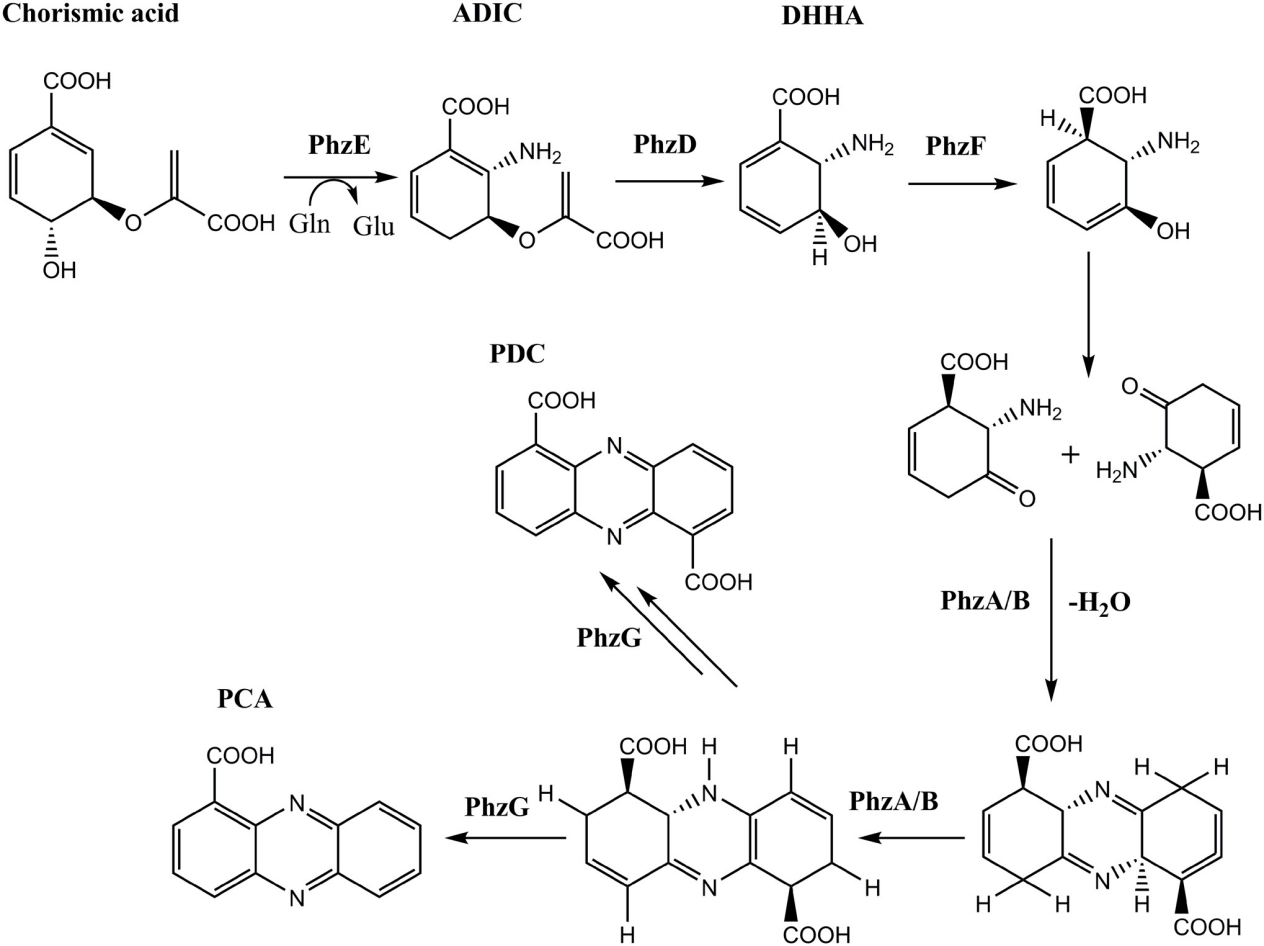
Proposed biosynthetic pathway of core phenazines[2,4,25].

Natural phenazines are biosynthesized via the shikimate pathway and alternate pathways. The shikimate pathway is believed to be primary pathway of phenazines biosynthesis. The synthesis involves chorismic acid conversion to phenazine-1,6-dicarboxylic acid and phenazine-1-carboxylic acid [22, 23] via 2-amino-2-desoxyisochorismic acid and *trans*-2,3-dihydro-3-hydroxyanthranilic acid (DHHA) [24]. Phenazine-1,6-dicarboxylic acid and phenazine-1-carboxylic acid are the precursors for more complex phenazine derivatives [2, 25]. McDonald and coworkers have systematically studied the enzyme PhzA-G [22], and partly elucidated the core phenazine biosynthetic pathway, as shown in Fig 1.

In this work, to clarify the key step in phenazine biosynthesis, we focused on the specific phenazine biosynthesis enzyme PhzF. PhzF is a representative of the enzyme family [26] and belongs to an operon containing the seven conserved genes *phzABCDEFG* responsible for assembly of the core phenazine tricycle by *Pseudomonas fluorescens* 2–79 and other fluorescent *Pseudomonas* spp. [1, 4, 27–30]. PhzF catalyzes the isomerization of its substrate DHHA; this is a crucial step in forming the basic phenazine aromatic structures such as phenazine-1,6-dicarboxylic acid and phenazine-1-carboxylic acid as shown in Fig 1. [31].

**Fig 2:**
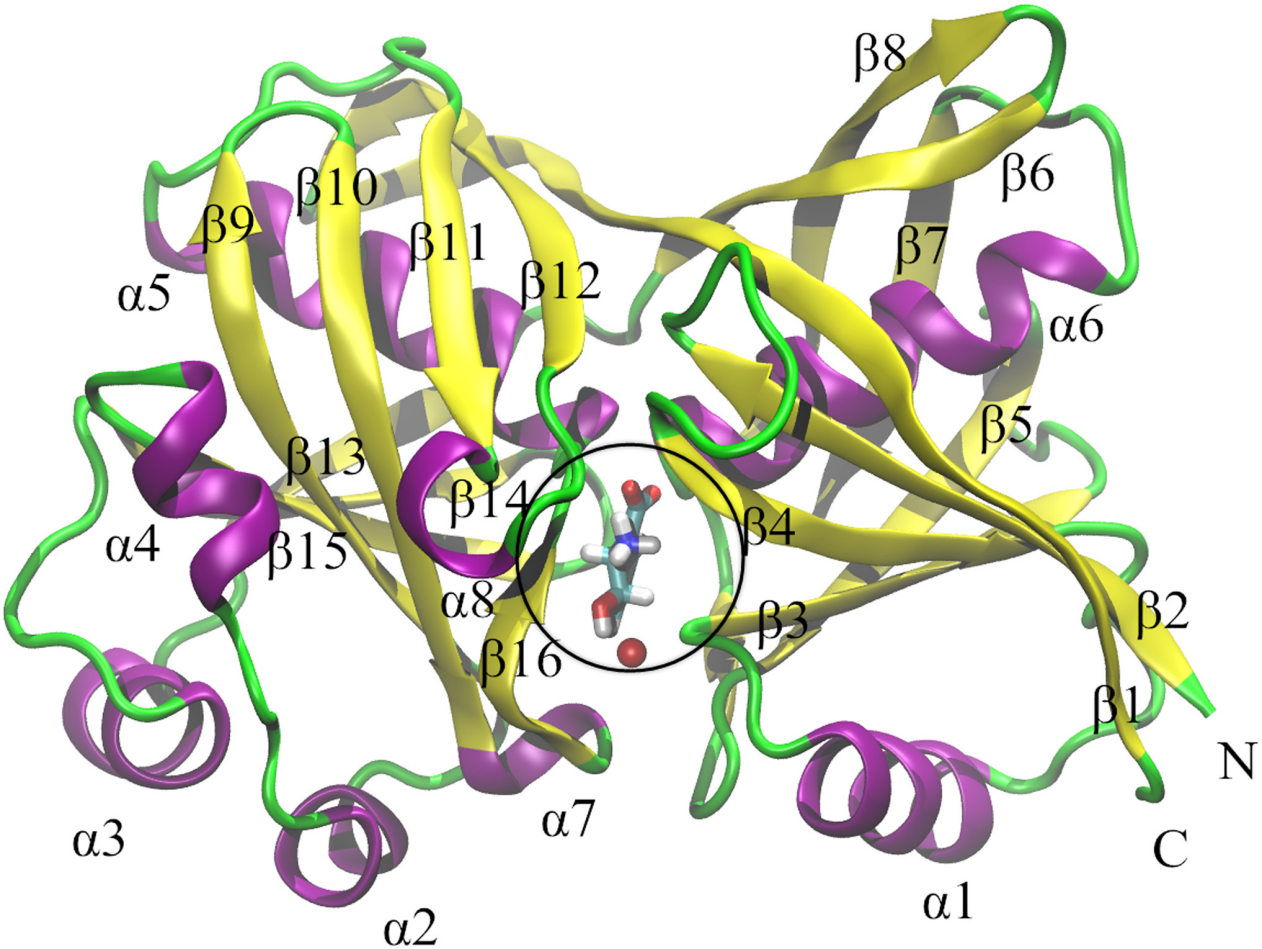
Single Subunit of PhzF and its Active Site. The single subunit of PhzF is displayed in NewRibbons style in VMD [33]; α-helix is colored violet (α1–α8); β-sheet is colored yellow (β1–β16); loop regions are colored green; the part in black circle is active site of PhzF.

PhzF is a symmetric homodimer of two subunits, consisting of 278 amino acids per subunit, with one active site. The single subunit consists of eight α-helixes, 16 β-sheets, and about 20 loop regions. Several residues contribute directly and indirectly to chemical reactions at the active site. The results of a large number of X-ray diffraction, nuclear magnetic resonance and other spectroscopic studies, and directed mutagenesis studies have shown that Glu45, His74, and Asp208 are conserved and are three catalytically critical residues [3, 26–28, 31]. Glu45 is the key residue and is responsible for proton transfer at active sites [31, 32]. Gly73, Ser213, and Thr211 have been shown to be essential for catalytic activity by hydrogen bonding with ligands [31]. Asp208 forms a hydrogen-bonded network with Asn18, Ser44, Ala210, Thr211, and the amino group of the ligand at the active site. Other residues near the active site, including Leu69, Ala72, His155, Ala183, Met198, Tyr203, and Val205, may provide hydrophobic contact with the ligand and help to maintain the correct conformation of the enzyme [31]. In addition to these residues, one water molecule at the active site can be involved to form hydrogen bonds with ligands. The catalytic activity is determined by a small number of active site residues, as shown in Fig 2.

**Fig 3:**
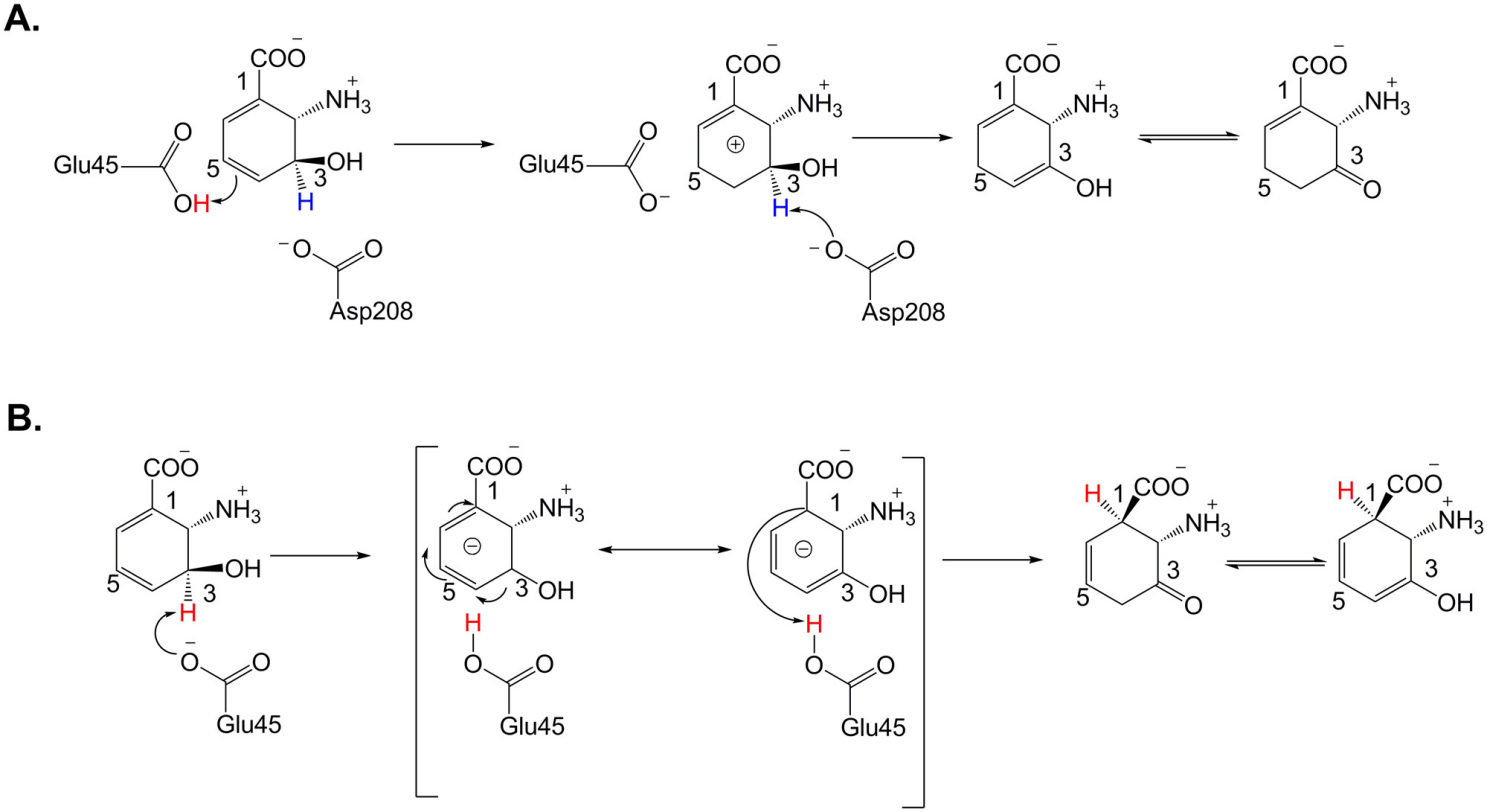
Proposed Mechanisms for Catalysis by PhzF [27, 31].

Two experimental studies of the catalytic mechanism of PhzF have been reported. The proposed mechanisms are shown in Fig 3 [22, 27, 31]. Parsons et al. first published the crystal structure of PhzF without a bound substrate, and proposed mechanism A in Fig 3 [27]. They suggested that the reaction starts with proton abstraction from protonated Glu45, a general acid, and then Glu45 donates a proton to C5 of DHHA, to form a carbocation at C4 of DHHA. Asp208, a general base, could abstract a proton from C3, tautomerize and form the 3-oxo product of DHHA. Subsequently, Blankenfeldt et al. determined the crystal structure of PhzF in a complex with its substrate DHHA and the product of DHHA’s isomerization. Based on their experimental data, they proposed a different catalytic mechanism B in Fig 3 [31]. In this mechanism, Glu45 acts as a general acid/base catalyst, transferring a proton from C3 to C1 of DHHA. Glu45 is ionized and withdraws a proton from C3 of DHHA, which leads to the formation of a dieanolate intermediate in which the negative charge is strongly delocalized along the O−C3−C4−C5−C6 conjugated system. This charge delocalization allows the transfer of a proton from the carboxyl group of Glu45 to C1. After proton transfer, Glu45 is ionized again, restoring the initial state of the enzyme, which is ready to accept a new substrate molecule. Some researchers in the field, e.g., Leland and Matthias, supported the mechanism proposed by Blankenfeldt et al. [2, 4]. Recently, Blankenfeldt and Parsons published results that confirm that mechanism B is appropriate for reactions catalyzed by PhzF [34]. Our computational studies were therefore performed based on mechanism B.

Detailed knowledge of the mechanism of reactions catalyzed by PhzF is of great importance in understanding its role. Despite all the efforts described above, the mechanism has still not been confirmed. Because of the relatively fast proton transfer, it is difficult to investigate the mechanism directly experimentally. Molecular simulation is an ideal method for dealing with such difficulties [35]. As far as we know, no computational investigation of the full catalytic process of PhzF has yet been published in the literature. In this paper, we present a computational study of the catalytic mechanism of PhzF, using molecular dynamics (MD) simulations and combined quantum mechanics and molecular mechanics (QM/MM) methods, aimed at understanding the detailed mechanism. MD and QM/MM are powerful tools and have been widely used in studies of enzyme-catalyzed reactions and mechanisms [36–38]. The goal in this work was to discern the details of the reaction process, which are valuable in clarifying the mechanism of PhzF catalysis, and to scrutinize the role of the active site residues in particular.

## Methods

### MD simulations

We performed MD simulations on four molecular systems: Apo enzyme (Apo), enzyme–substrate complexes with negatively charged Glu45 (ES), enzyme–transition state analog inhibitor complexes with neutral Glu45 (ET), and enzyme–product complexes with negatively charged Glu45 (EP). The PhzF homodimer with DHHA ligands was downloaded from the published crystal structure in the Protein Data Bank (PDB ID: 1U1X, 1.88 Å resolution) [31]. MD simulation of the PhzF transition state was performed using the coordinates of PhzF bound with a transition state analog inhibitor, 3OHAA (PDB ID: 1U1W, 1.35 Å resolution) [31]. Chain A of the dimer was used to provide the starting coordinates for the simulations. The ES system was solvated in a rectangular water box of size 80 × 80 × 100 Å with TIP3P water molecules [39], using the center of mass of the protein as the coordinate of the origin, and periodic boundary conditions [40] (Table A and Fig A in S1 File). Na^+^ and Cl^−^ ions were added to neutralize the system. Similar strategies were used to model the Apo, ET, and ES systems (Table A in S1 File). The systems to be simulated were first subjected to conjugate gradient minimization and equilibrated for 500 ps under NVT ensemble conditions to relax the systems. The simulations were then continued for 200 ns under NPT ensemble conditions, without restraints, at a constant pressure of 1 bar and a constant temperature of 310 K. A time step of 2 fs was used in the MD simulations [41], and the trajectory data were saved at 200 ps intervals. The cutoff for short-range and nonbonded interactions was calculated at 12.0 Å. The long-range electrostatic interactions were obtained using the particle mesh Ewald algorithm [42]. All bonds involving hydrogen atoms were constrained using the SHAKE algorithm [43]. All MD simulations were performed using NAMD 2.9 [44] software with the CHARMM27 force field [45], and all dynamic trajectories were analyzed using the VMD [33] and GROMACS programs [46]. MD simulations ensured the stability of the protein structure, and its coordinates were used to build the starting structure of the truncated active site for the QM/MM computations. A schematic diagram of DHHA bound to the PhzF binding site is shown in Fig 2. The p*K*_a_ values for residues at pH 7.4 (physiologic) were calculated using the PROPKA3.1 program [47, 48], and the Glu45 and Asp208 residues were deprotonated according to their polar environment (Table B in S1 File).

#### Principal component analysis

Principal component analysis (PCA) is a useful tool for converting a set of correlated variables to uncorrelated ones called principal components [49]. In this work, PCA was performed to identify the most significant fluctuation modes of the proteins by simplifying the number of motional dimensions. The eigenvectors and eigenvalues were based on calculation of diagonalization of the covariance matrix. For our simulations, all backbone heavy atoms were determined using PCA [50]. All PCA analysis was performed using GROMACS [51, 52].

#### QM/MM calculations

The reaction mechanism of PhzF was investigated using QM/MM calculations [35, 36, 53, 54]. The starting geometries of the reactant, transition state 2 (TS1), intermediate, transition state 2 (TS2), and product models consisting of 360 atoms, were cut off from the MD structure simulations (Fig A in S1 File). In the QM/MM calculations, the truncated active site was divided into two layers: an inner layer (QM region) and outer layer (MM region) [54, 55]. The QM region, which involves the formation and cleavage of chemical bonds, was described using density functional theory [56]; and the MM region, which represents the surrounding environment of the QM region, was treated using the universal force field [57, 58]. The mechanical embedding scheme was used for treating the interactions between the QM and MM systems. The substrate and part of the residue were included in the QM region, which contained 29 atoms; the rest of system was included in the MM region (Fig C in S1 File). In the QM/MM calculations, only atoms surrounding the substrate were allowed to move freely; the remaining atoms in the system were frozen (Fig B in S1 File). The total charge of the QM region was −1 and the spin multiplicity was 1. Hydrogen atoms were used as linking atoms to saturate the dangling bonds between the inner layer and the outer layer. The QM region was treated using density functional theory with the B3LYP function, which is the most widely used function in dealing with various chemistry problems [59, 60]. For the QM region, geometry optimizations were performed at the B3LYP/6-31G (d,p) level, and single-point calculations were performed using the 6–31+G (d,p) basis set [61, 62]. All QM/MM calculation were performed using the Gaussian09 package [63]. The transition-state structure was determined by scanning over the bond lengths to find transition states. The activation energy barriers of the reaction, reactant complex, transition state, intermediate, and product complex structures were optimized geometrically.

## Results and Discussion

### MD simulations

**Fig 4:**
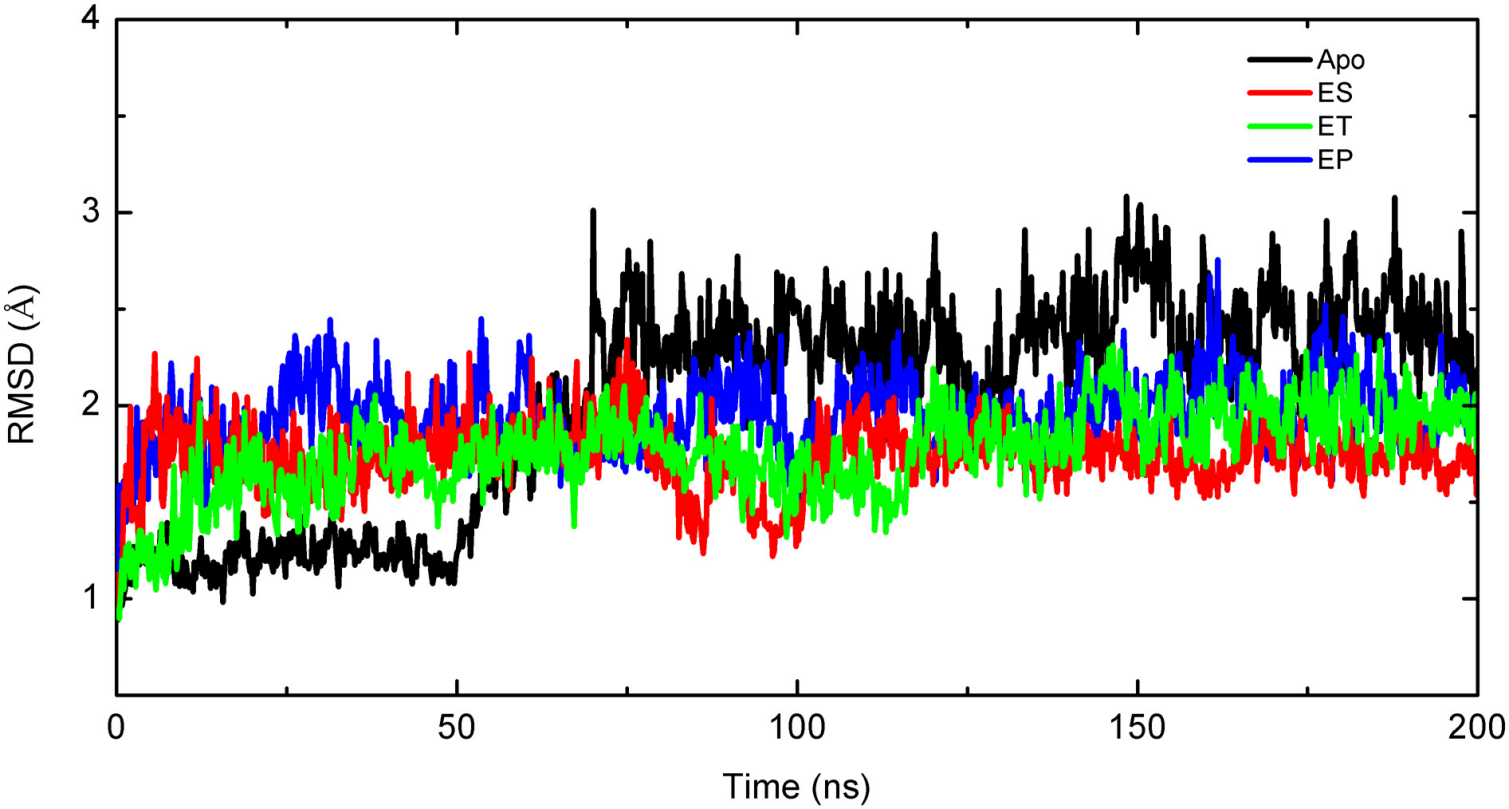
RMSDs of Backbone for Apo (black), ES (red), ET (green), and EP (blue).

We performed 200 ns simulations on the Apo, ES, ET, and EP systems. The root-mean-square deviations (RMSDs) of the entire backbone during the simulation time showed that the backbone trajectories were stable, reaching equilibrium after the first 500 ps of the simulation, as shown in Fig 4. In the EP, ES, and ET systems, which showed similar trends, there was a rapid rise in the first 10 ns of the simulation, and then the values remained stable during the last 190 ns of simulation. The average RMSD values for the EP, ES, and ET are 1.84 ± 0.20 Å, 1.74 ± 0.20 Å, and 1.76 ± 0.23 Å, respectively. For the Apo system, the simulation experienced two equilibrations. After the first 10 ns of simulation, the RMSD curves of Apo reached a first plateau, with an average value of 1.64 ± 0.23 Å. After simulation for 50 ns, the Apo RMSD curves reached a second plateau, with an average value of 1.95 ± 0.20 Å. The RMSD curves of Apo followed the same trend as those for EP, ES, and ET throughout the simulations. The RMSD of the Apo is slightly higher than those of the EP, ES, and ET, indicating that there is more structural flexibility. In the MD simulations of the ES, ET, and EP complexes, water molecules were observed to have similar behaviors, and they escaped from the active site during the simulation time. For all the simulations, from 10 ns until the end of the simulation, the values fluctuated very little, indicating equilibrated protein structures.

**Fig 5:**
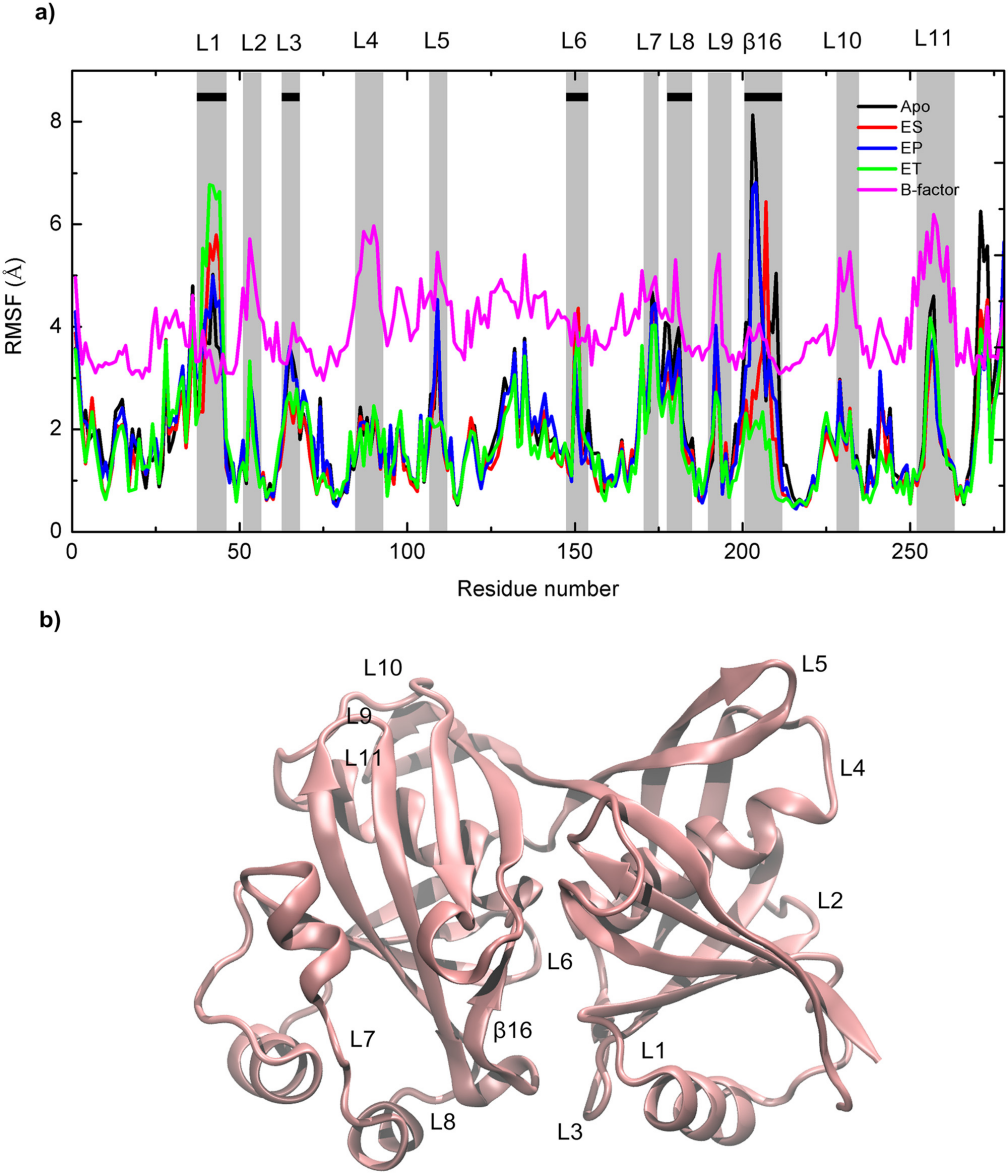
RMSF Values of Residues for Apo (black), ES (red), ET (green), and EP (blue) (a) and with Respect to Crystal Structure (b). Gray vertical bars show regions with high RMSF values. Second structures surrounding active site are shown by black horizontal bars.

The root-mean-square fluctuations (RMSFs) for the Apo, ES, ET, and EP systems were obtained; representative results are shown in Fig 5a and 5b. It was expected that the residues of the active site region, loop region (including coils and turns), and N- and C-termini would have high RMSF values, and this was proved by our analysis to be correct for all the simulated systems. The high RMSF values mean that these regions have high flexibilities. The active site domain was surrounded by regions with high RMSF values, including one β-sheet β16 (residues 203–208) and four loop regions, i.e., L1 (residues 41–45), L3 (residues 66–68), L6 (residues 150–153), and L8 (residues 180–183), represented by black horizontal bars in Fig 5a. The other regions that had high RMSF values located on loop regions were L2 (residues 52–55), L4 (residues 87–91), L5 (residues 108–110), L7 (residues 170–174), L9 (residues 192–193), L10 (residues 229–232), and L11 (residues 255–259), represented by gray vertical bars in Fig 5a.

### Passage leading to active site

Previous studies did not report passages leading to the active site, which can act as channels for small molecules such as reactants, products, water, and ions. In this study, we investigated the dynamic properties of passages based on the MD simulations.

**Fig 6:**
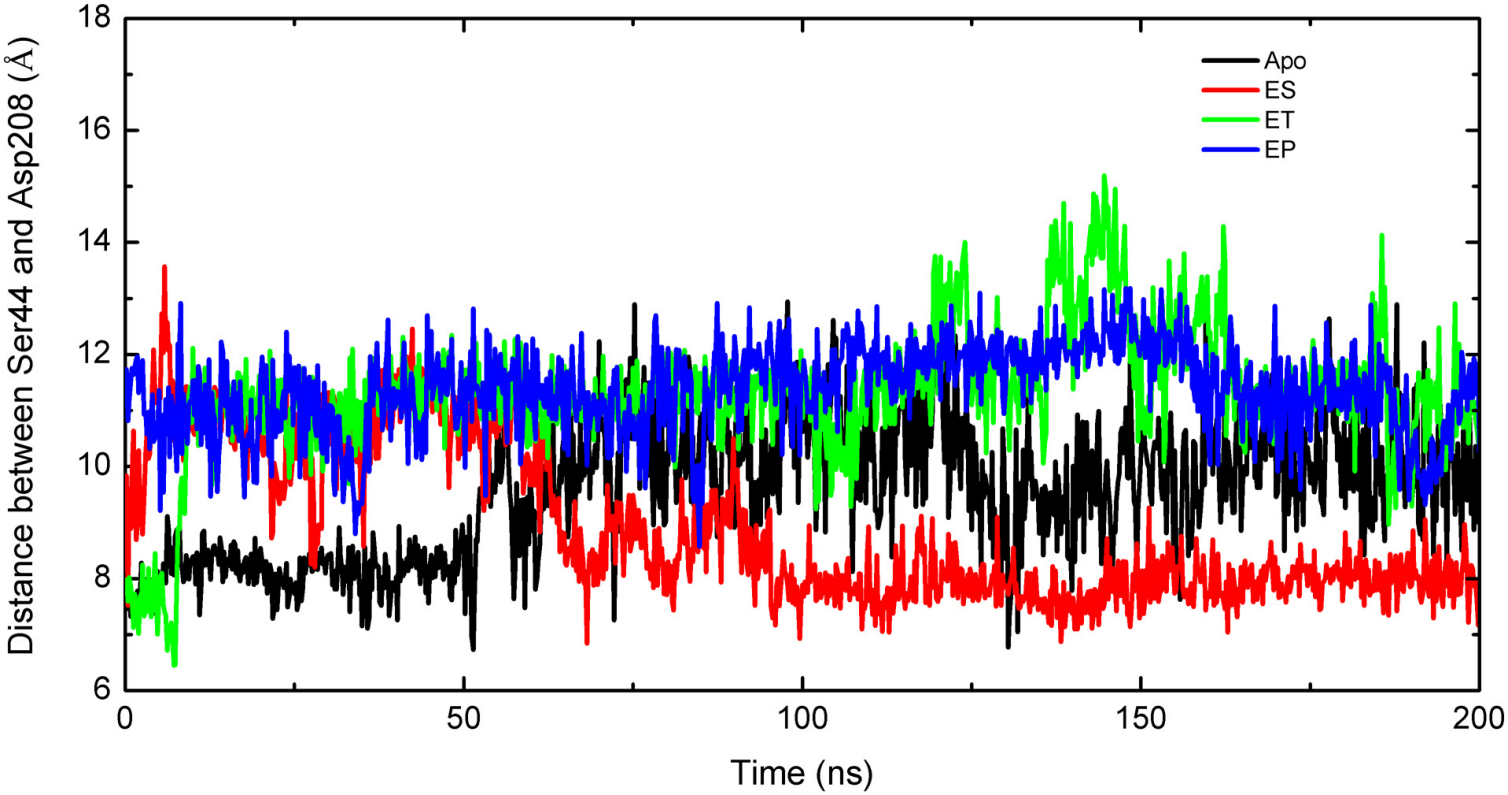
Fluctuations in Distances between Residues Ser44 and Asp208 for Apo (Black), ES (Red), ET (Green), and EP (Blue).

Analysis of the MD trajectory shows that the structures of β16 (residues 203–208) and L1 (residues 41–45) were highly flexible over the course of the simulations. The crystal structures show that these two structures were located at two sides of the active site pocket entrance, acting as a door to control the passage width. The door consisted of residues Met41, Asn42, Leu43, Ser44, and Glu45 on one side, and residues Tyr203, Gly204, Val205, Val206, Glu207, and Asp208 on other side of the PhzF pocket. In addition, Ser44 and Asp208 were key residues at two sides of the door. To determine the dynamic features of the active site entrance, the minimum/maximum distances between Ser44 and Asp208 (S–D distance) were monitored during the MD simulations. The fluctuations in the S–D distances during the 200 ns MD simulations for the four systems are shown in Fig 6.

The entrance to the active site of the ES complex clearly closed after simulation for about 90 ns, and the S–D distances generally decreased during the MD simulations. The S–D distance of the ET complex fluctuated slightly around 8 Å from 0 to 10.0 ns; at ∼10.0 ns, the S–D distance suddenly increased to ∼12 Å (Fig 6), and the active site remained open until the end of the simulation. The EP complex behavior was similar to that of ET, and it maintained an open state during the entire simulation. The results show that the close/open state of the entrance correlated with rotation of Glu45 by 172.75° (this will be discussed later in the section on QM/MM calculations). As a consequence, the hydrogen bond between Glu45 and $2'-{\text{NH}}_{3}^{+}$ was broken. Once this hydrogen bond was lost, the closed state was not regained, therefore the entrances to the ET and EP complexes were able to remain open.

### Principal component analysis

**Fig 7:**
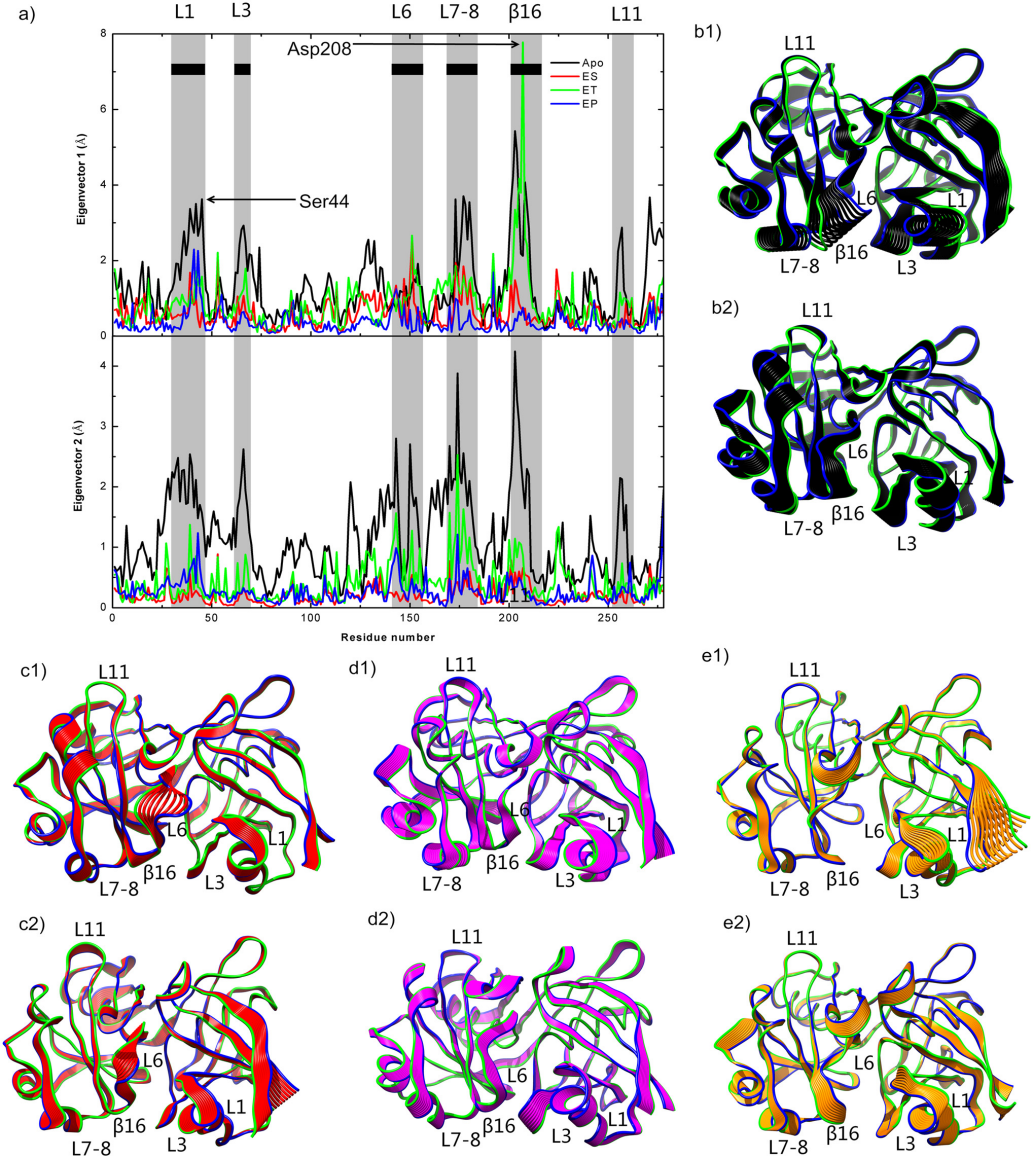
Values for the first and second Eigenvectors Obtained from PCA on MD Simulations. (a) RMSF values for backbone atoms in first and second eigenvectors of Apo (black), ES (red), ET (green), and EP (blue). Gray vertical bars show regions with high RMSF values. The second structures surrounding the active site are shown by black horizontal bars. (b), (c), (d), and (e) The 10 sequential frames representing extension of the fluctuations of the protein backbone atoms along first and second eigenvectors after projection of the trajectory of Apo (black), ET (red), ET (violet), and EP (yellow) on the corresponding eigenvectors. The first extreme is shown in blue and the last extreme in green.

PCA was performed based on the MD simulation results for the Apo, ES, ET, and EP complexes, to analyze the conformational changes and compare the structural features. The first two eigenvalues of the principal components for the Apo, ES, ET, and EP complex accounted for approximately 84%, 78%, 80%, and 79% of the total motions, respectively. Fig 7a shows the RMSF values of the backbone atoms in the first and second eigenvectors of Apo (black), ES (red), ET (green), and EP (blue). The corresponding snapshots extracted from the projection of the first and second eigenvectors on MD trajectories are shown in Fig 7b–7e for Apo, ES, ET, and EP, respectively. In all the simulations, the residues of the loop regions (L1, L3, L6, L7, L8, and L11) and β16 showed higher fluctuations in the first eigenvector and in the second eigenvector. In both the first and second eigenvectors, the Apo fluctuations were higher than those of ES, ET, and EP. ET showed higher fluctuations in β16 in the first eigenvector, but were not in the second eigenvector. Our analysis confirms that two key residues, i.e., Ser44 and Asp208, act as a door to control the pocket entrance (closed and open states). ES had lower fluctuations during the simulations and tended to have a closed entrance; ET had higher fluctuations, and tended to have an open entrance.

### QM/MM calculations

**Fig 8:**
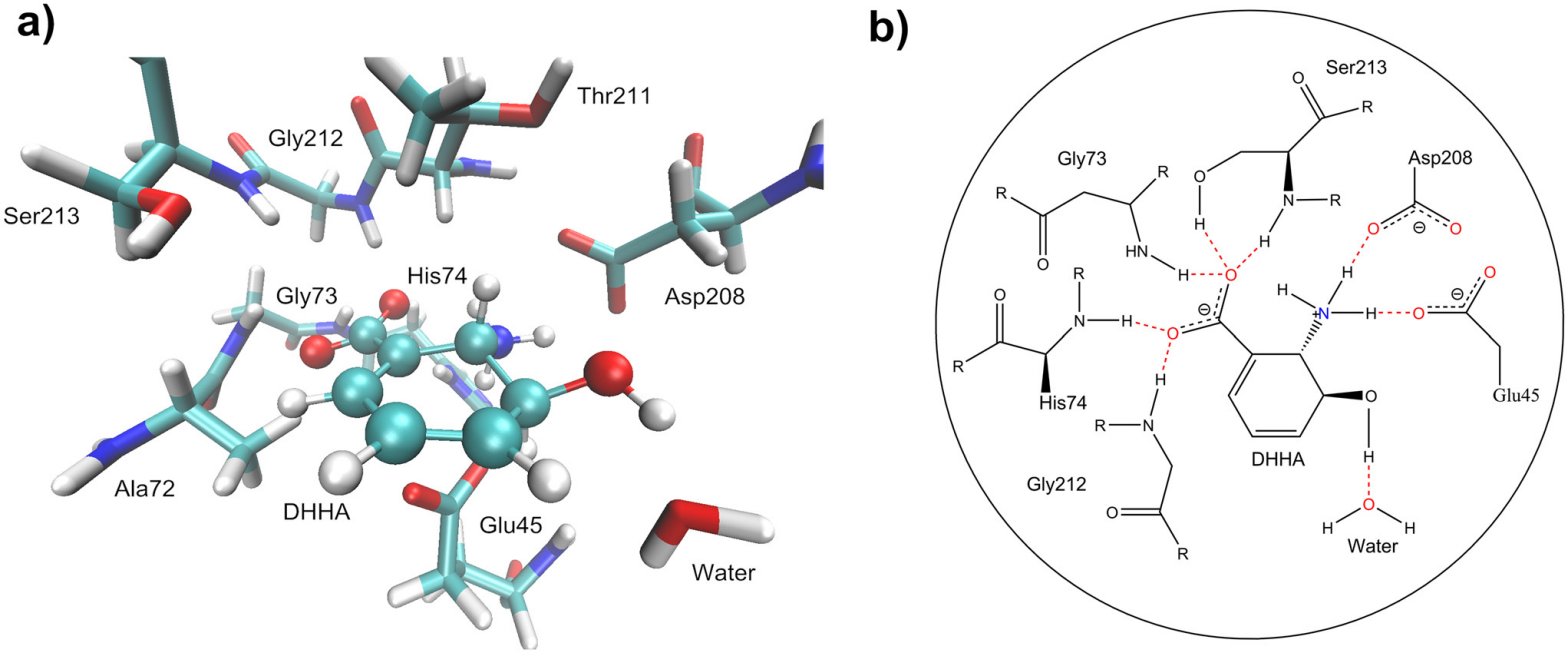
Stereoview of DHHA Binding to Active Site of PhzF and Hydrogen-bonding Interactions between DHHA and Active Site Residues. (a) DHHA is shown in CPK style; water molecule and side chains of active site residues in close proximity to DHHA are shown in Licorice style. (b) Hydrogen-bonding interactions between DHHA and active side residues are shown in red.

The importance of Glu45 for enzymatic activity is well known [31]. However, its precise function remains unclear. In the present work, QM/MM calculations were performed for reactant, TS1, intermediate, TS2, and product models. These models were optimized using Gaussian 09 with the default convergence criteria. The goal of these studies was to elucidate the mechanism of catalysis by PhzF and to define the role of the individual active site residues at an atomic level. The models presented here indicate that unprotonated Glu45 was protonated prior to proton transfer, and acted as general acid/base. These models suggest that Gly73, His74, Asp208, Gly212, and Ser213 formed hydrogen bonds with the substrate to stabilize proton transfer. The catalytic roles of the conserved active site residues are shown in Fig 8.

**Fig 9:**
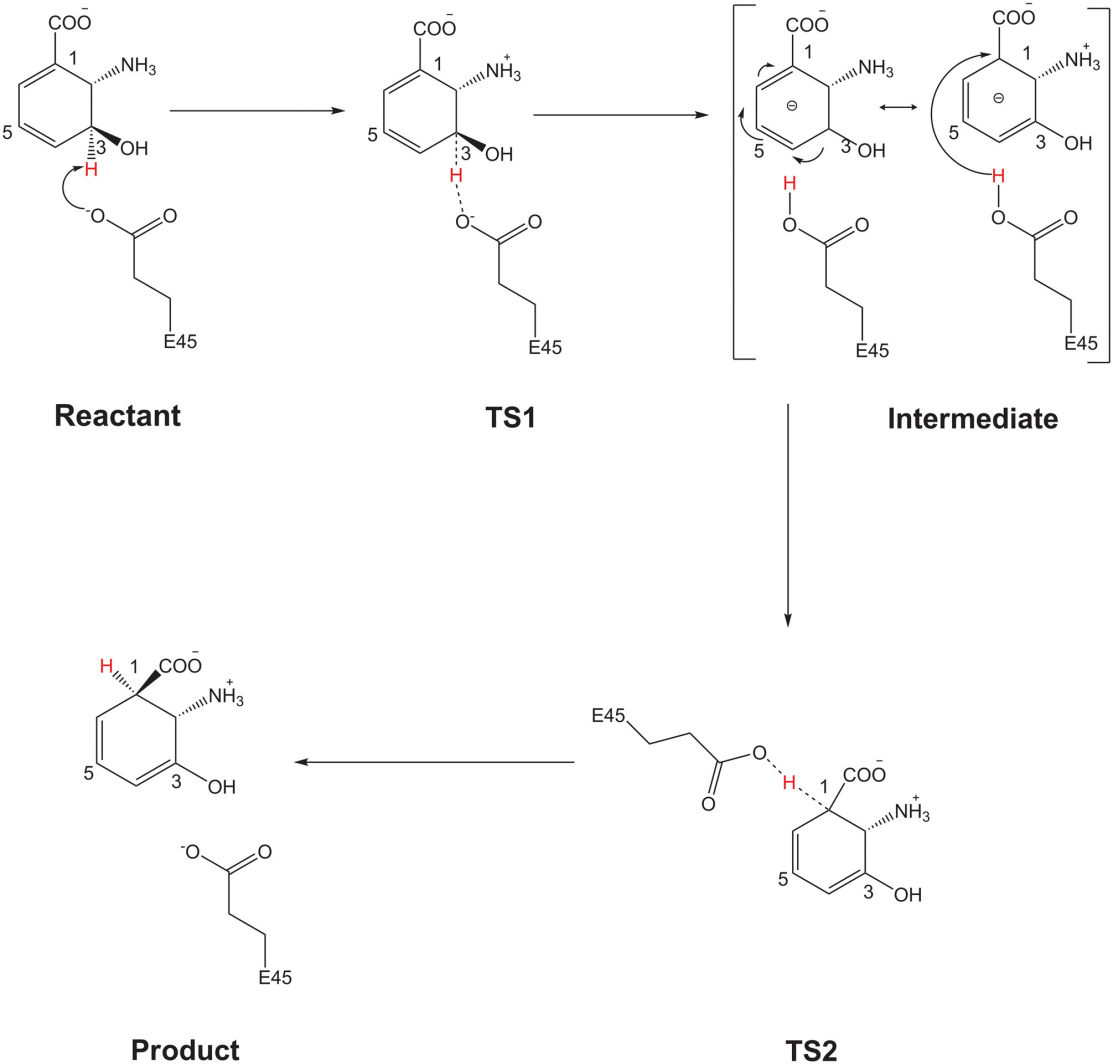
Reaction Path for Catalytic Mechanism.

The catalytic mechanism of PhzF is shown in Fig 9. Starting from the noncovalent complex, the Glu45 carboxyl group is positioned to create an electrostatic interaction with C3 of DHHA. In Step 1, the oxygen of Glu45 attacks and abstracts the C3 proton, resulting in formation of an intermediate involving TS1. In Step 2, the Glu45 donates the proton to C1 of DHHA, forming a product involving TS2.

### Reactants

As noted in the introduction, the previously proposed mechanism B involved proton transfer and rearrangement of the double-bond system. DHHA was surrounded by Glu45, Gly73, His74, Asp208, Gly212, and Ser213, which were responsible for creating the hydrogen-bonding interactions (Fig 8). The water molecule established a hydrogen bond with DHHA at a distance of 2.35 Å. The key residues, Glu45 and Asp208, at the active site were unprotonated. Glu45 and Asp208 each contained a negatively charged carboxylic group, and stabilized the protonated amino group of DHHA via electrostatic and hydrogen-bonding interactions, with distances of 1.54 and 2.49 Å, respectively. The backbone amide moieties, Gly73, His74, Gly212, and Ser213, formed hydrogen bonds with carboxylic group oxygens, and OH_Ser213_ formed a bond with the carboxylic group oxygen, which also helped to stabilize the conformation of the active site.

### First reaction step–Glu45 acting as base

The first step in this catalytic mechanism involved breakage of the C3–H bond of DHHA, and formation of an intermediate. In our calculations, the $2'-{\text{NH}}_{3}^{+}$ group formed hydrogen-bonding interactions with Asp208 (with a distance of 2.43 Å) and Glu45 (1.81 Å). The length of the hydrogen bond between 3'-OH and water was 2.40 Å. The 1'-COO^−^ established hydrogen bonds with the Ser213 side chain hydroxyl group (2.59 Å), the His74 imino group (2.62 Å), the Gly212 imino group (2.40 Å), and the Gly73 imino group (2.23 Å). Some of these hydrogen bonds were very short and played important roles in stabilizing the transition state via their hydrogen-bonding interactions with 1'-COO^−^, $2'-{\text{NH}}_{3}^{+}$ and 3'-OH, respectively. Blankenfeldt’s studies suggested that these hydrogen-bonding interactions were important for substrate binding and stabilization of the transition states in the catalytic process [31]. Our calculations were in agreement with their suggestions.

Full optimization of the TS1 structure and vibrational frequency analysis of TS1 showed that there was just one imaginary frequency (1243 cm^−1^), in agreement with the reaction coordinate in first step of the reaction. Analysis of TS1 shows that breaking of the C3–H bond occurred, with protonation of Glu45. The O_Glu45_–H distance was 2.10 Å in the reactant, and decreased to 1.20 Å in TS1 (Fig D in S1 File). When the carboxylic group of Glu45 came close to the proton on C3, the proton was attracted and transferred to the carboxylic oxygen of Glu45, resulting in protonation of Glu45. Subsequently, rearrangement of the double-bond system of the ring was observed in the transition state. In this step, the double-bond system was completely delocalized along C3−C4−C5−C6−C1, as clearly indicated by the C−C distances (C3−C4, C4−C5, C5−C6, and C6−C1 were 1.35, 1.44, 1.37, and 1.42 Å, respectively). The hybridization of C1 changed from sp2 to sp3, which contributed to the formation of a C1 anion.

At the end of this step, the intermediate structure was identified. The O_Glu45_–H distance decreased to 1.01 Å (Fig D in S1 File), indicating that protonation of Glu45 had occurred. The carboxylic group of the protonated Glu45 rotated toward the C1 anion through an angle of 172.75°, oriented itself toward the carbanion, and then established electrostatic interactions. In the intermediate geometry, the hydrogen-bond distances did not change significantly in comparison with those in TS1, indicating that the roles of the residues around the active site were merely to maintain intermediate stabilization.

### Second step–Glu45 acting as acid

In the second mechanistic step, Glu45 donated its proton to the C1 anion and formed a product. We optimized the TS2 structure and analyzed the vibrational frequencies. The results show that an imaginary frequency (809.73 cm^−1^) in TS2 for proton transfer corresponded to the hydrogen atom vibration between the Glu45 carboxyl oxygen and C1. The optimization provided the correct position for Glu45 to approach the C1 anion, enabling an electrostatic interaction with the C1 anion to be established.

Gly73, His74, Asp208, Gly212, and Ser213 were important for TS2 stabilization by hydrogen-bonding interactions with the substrate. The stabilizing interactions for the TS2 structure were similar to those observed for TS1. However, the distances between the $2'-{\text{NH}}_{3}^{+}$ group and the Glu45 in the reactant (1.54 Å) and product (1.43 Å) were different to those in TS1 (1.81 Å), the intermediate (1.82 Å), and TS2 (1.78 Å); this was caused by back and forth rotation of the Glu45 carboxyl group during the catalytic process. The hydrogen-bonded network described above was still working to stabilize the active site in the product structure. The H–C1 distance was 1.53 Å in TS2, and decreased to 1.10 Å in the product (Fig D in S1 File).

The active site complexes created special electrostatic environments and maintained specific conformations that were suitable for proton transfer. The protonated Glu45 was attracted by C1 and rotated away from C3 to C1. The proton of the Glu45 carboxyl group was then pointed toward C1 and formed a new electrostatic interaction with it. Subsequently, double-bond rearrangement was accomplished in the product, as clearly indicated by the C−C distances (C3−C4, C4−C5, C5−C6, and C6−C1 distances were 1.34, 1.47, 1.34, and 1.49, Å, respectively). When Glu45 donated a proton to C1, the electrostatic interactions repulsed negatively charged Glu45 back to its initial position, preparing it for another reaction cycle (S1 Video).

**Fig 10:**
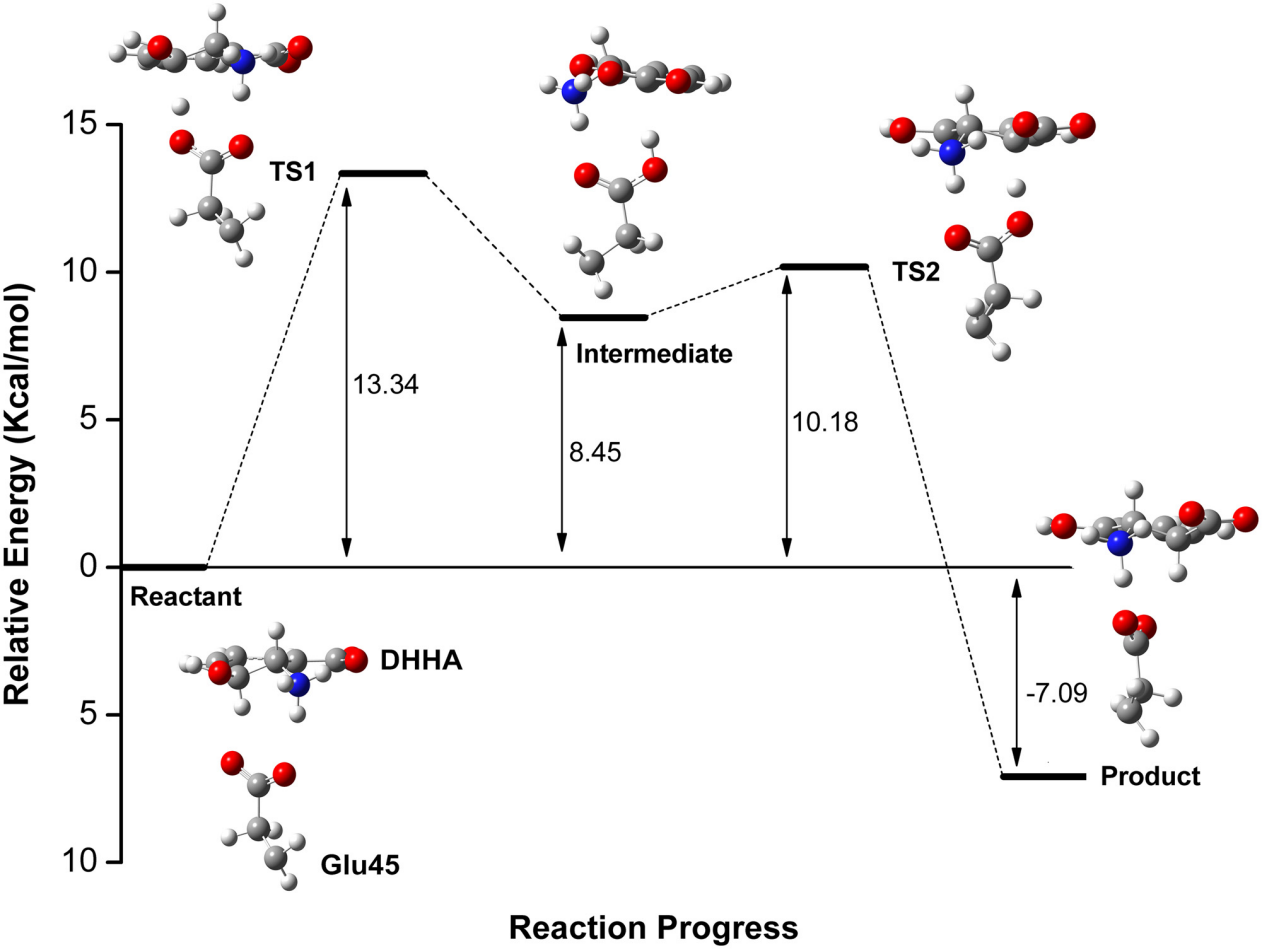
Energy Profile for Catalytic Process.

As mentioned above, ONIOM approaches provided a new view of the reaction mechanism. Fig 10 shows the reaction energy profile for the proton transfer isomerization reactions. The energy profile shows that the isomerization reaction was a two-step mechanism involving TS1 (with an energy barrier of 13.34 kcal mol^−1^) and TS2 (with an energy barrier of 10.18 kcal mol^−1^). In the former transition structure, the Glu45 deprotonated C3 of the reactant, forming an intermediate; in the latter, Glu45 released its carboxyl proton to C1, yielding the product.

## Conclusion

In this study, we performed a detailed computational investigation of PhzF using QM/MM and MD simulations, to understand the enzymatic isomerization reaction mechanism. During the simulations, the backbone atoms of Apo, ES, ET, and EP were observed to have flexibilities similar to that of the initial crystal structure. The simulations showed high fluctuations of the structures of β16 and L1, which form a passage leading to the active site, which was not observable from static crystal structures [27, 31].

In a previously proposed mechanism, it was suggested that the Glu45 residue abstracted the proton at C3 of the substrate to initiate the isomerization reaction. We built the reactant, TS1, intermediate, TS2 and product models to confirm this process. Our results proved that Glu45, acting as a general acid/base, is involved in proton transfer from C3 to C1. Our models revealed double roles of Glu45 in the isomerization reaction: it not only formed a hydrogen bond with the amino group at C2 of the substrate to help the enzyme bind and recognize the substrate, but also initiated the isomerization reaction by behaving as a proton shuttle.

In addition, our models account for the important hydrogen-bonding network. We found that Asp208 did not participate directly in proton transfer but was hydrogen bonded with the amino group at C2 of the substrate. A water molecule was hydrogen bonded with the hydroxyl group at C3 of the substrate, and the residues Gly73, His74, Gly212, and Ser213 formed hydrogen bonds with carboxylic group oxygens, which helped to bind and stabilize the structure at the active site.

Our results strongly support mechanism B in Fig 3. This study provides a deeper understanding of the structure and function of PhzF and will enable rational modification of the enzyme to improve the catalytic efficiency.

## Supporting Information

S1 File Supporting tables and Figs. Summary of the simulations used for the Apo, ES, ET, and EP systems (**Table A**). Protonation state of three key amino acids of PhzF at pH 7.4 (**Table B**). Representation of the system used for the simulations (**Fig A**). Representation of the free and frozen atoms used for QM/MM calculations (**Fig B**). ONIOM partioning of active site (**Fig C**). Representation of the stationary structures found from exploration of the potential energy surface corresponding to the proton transfer reaction (**Fig D**).
(DOCX)

S1 Video Motions of catalytic mechanism of PhzF. (AVI)
