# Supplementary material for: Elucidation of Enzymatic Mechanism of Phenazine Biosynthetic Protein PhzF Using QM/MM and MD Simulations
Source: PLoS One. 2015 Sep 28;10(9):e0139081. doi: 10.1371/journal.pone.0139081 (PMC4586147; doi:10.1371/journal.pone.0139081)
Supplement: S1 File — Summary of the simulations used for the Apo, ES, ET, and EP systems (Table A). Protonation state of three key amino acids of PhzF at pH 7.4 (Table B). Representation of the system used for the simulations (Fig A). Representation of the free and frozen atoms used for QM/MM calculations (Fig B). ONIOM partioning of active site (Fig C). Representation of the stationary structures found from exploration of the potential energy surface corresponding to the proton transfer reaction (Fig D). (DOCX) [file pone.0139081.s001.docx]

S1 Supporting Information

Table A. Summary of the simulations employed for the Apo, ES, ET and EP systems.

| System | Number of Atoms | Number of Na^+^ ions | Number of Cl^-^ ions | Total Simulation Time |
| --- | --- | --- | --- | --- |
| Apo | 45697 | 47 | 39 | 200 ns |
| ES | 46469 | 49 | 40 | 200 ns |
| ET | 47369 | 54 | 45 | 200 ns |
| EP | 45864 | 48 | 39 | 200 ns |

Table B. Protonation state of three key amino acids of PhzF at pH 7.4.

| Amino acids | *pK*a |
| --- | --- |
| Glu45 | 6.43 |
| His74 | 8.29 |
| Asp208 | 1.56 |


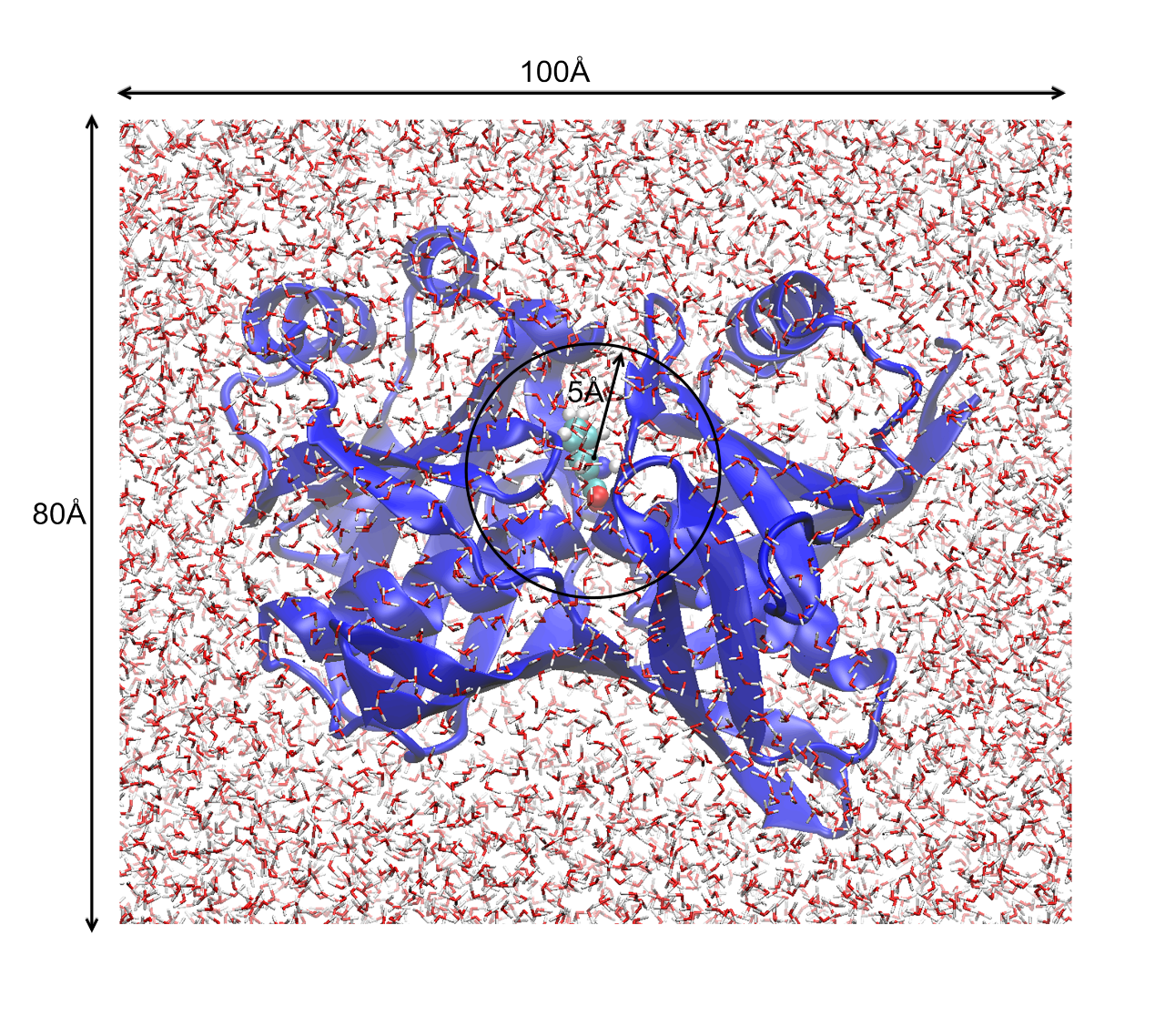


**Figure A. Representation of system used for the simulations.** QM/MM subsystem is shown in the black circle.

**
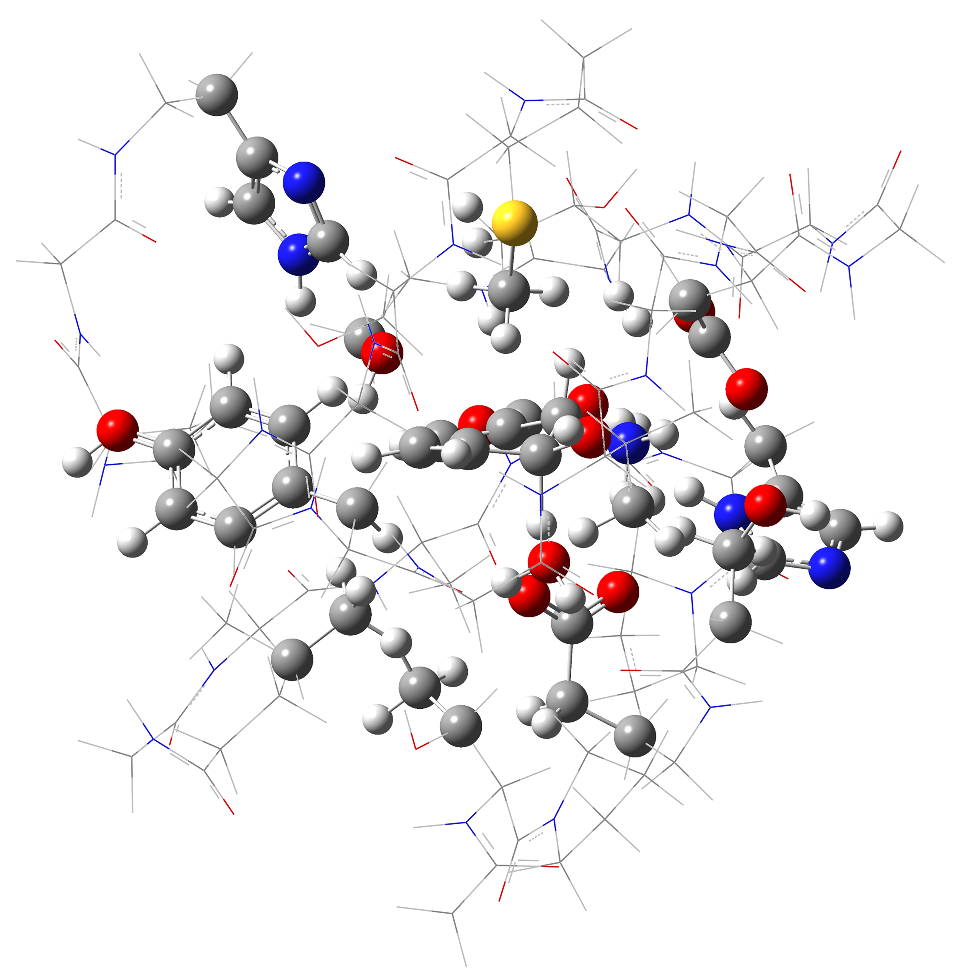
**

**Figure B. Representation of the free and frozen atoms used for QM/MM calculations.** The free atoms is shown in Ball and Bond style, the frozen atoms in Wireframe style.

**
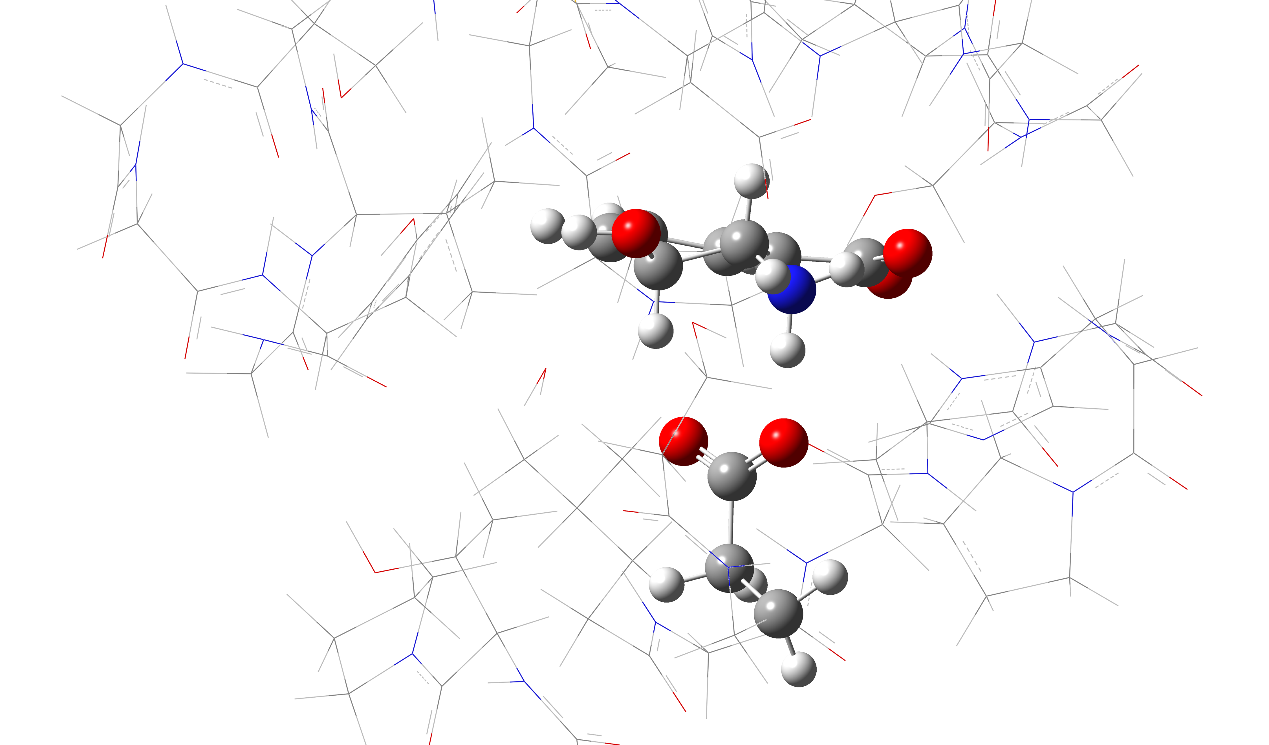
**

**Figure C. ONIOM partitioning of active site.** QM region is shown in Ball and Bond style, MM region in Wireframe style.

**
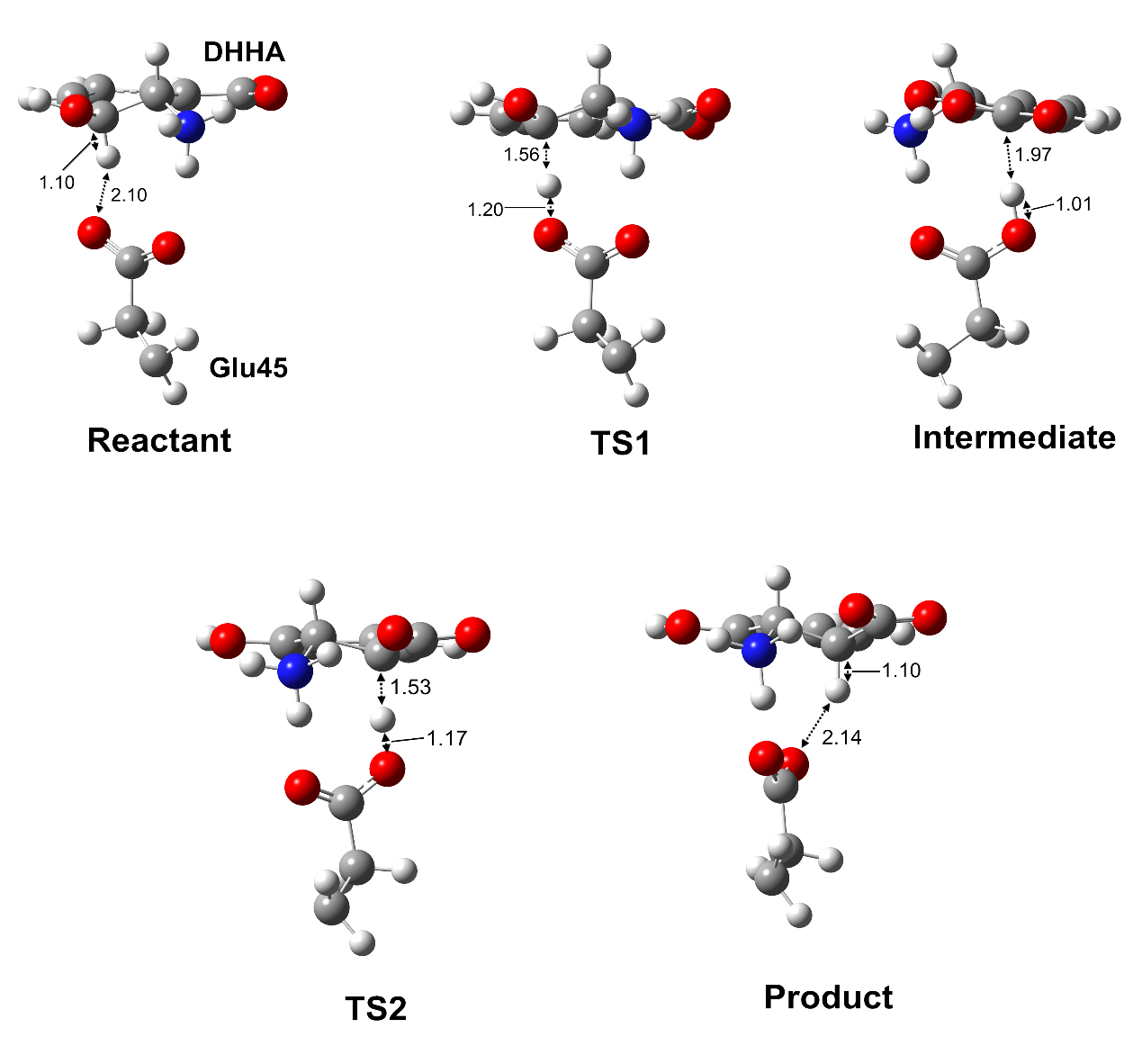
**

**Figure D. Representation of the stationary structures found from the exploration of the Potential**

**Energy Surface corresponding to the proton transfer reaction.** Distances in Angstroms.
